# An experimental method for estimating the tearing energy in rubber-like materials using the true stored energy

**DOI:** 10.1038/s41598-021-95151-y

**Published:** 2021-08-10

**Authors:** Elsiddig Elmukashfi

**Affiliations:** 1 Department of Solid Mechanics, Royal Institute of Technology, Teknikringen 8D, 114 28 Stockholm, Sweden; 2 Department of Engineering Science, University of Oxford, Parks Road, Oxford, OX1 3PJ UK

**Keywords:** Materials science, Physics, Engineering, Mechanical engineering

## Abstract

A method for determining the critical tearing energy in rubber-like materials is proposed. In this method, the energy required for crack propagation in a rubber-like material is determined by the change of recovered elastic energy which is obtained by deducting the dissipated energy due to different inelastic processes from the total strain energy applied to the system. Hence, the classical method proposed by Rivlin and Thomas using the pure shear tear test is modified using the actual stored elastic energy. The total dissipated energy is evaluated using cyclic pure shear and simple shear dynamic experiments at the critical stretch level. To accurately estimate the total dissipated energy, the unloading rate is determined from the time the crack takes to grow an increment. A carbon-black-filled natural rubber is examined in this study. In cyclic pure shear experiment, the specimens were cyclically loaded under quasi-static loading rate of $0.01~{\rm {s}}^{-1}$ and for different unloading rates, i.e. $0.01$, $0.1$ and $1.0~{\rm {s}}^{-1}$. The simple shear dynamic experiment is used to obtain the total dissipated energy at higher frequencies, i.e. $0.5$-$18~{\rm {Hz}}$ which corresponds to unloading rates $0.46$-$16.41~{\rm {s}}^{-1}$, using the similarities between simple and pure shear deformation. The relationship between dissipated energy and unloading stretch rate is found to follow a power-law such that cyclic pure shear and simple shear dynamic experiments yield similar result. At lower unloading rates (i.e. ${\dot{\lambda }}_{\rm {U}} < 1.0~{\rm {s}}^{-1}$), Mullins effect dominates and the viscous dissipation is minor, whereas at higher unloading rates, viscous dissipation becomes significant. At the crack propagation unloading rate $125.2~{\rm {s}}^{-1}$, the viscous dissipation is significant such that the amount of dissipated energy increases approximately by $125.4\%$ from the lowest unloading rate. The critical tearing energy is obtained to be $7.04~{\rm {kJ}}/{\rm {m}}^{2}$ using classical method and $5.12~{\rm {kJ}}/{\rm {m}}^{2}$ using the proposed method. Hence, the classical method overestimates the critical tearing energy by approximately $37.5\%$.

## Introduction

The tearing energy, as a fracture mechanics concept, was proposed by Rivlin and Thomas^1^ as an analogy to the energy release rate^2^ to study fracture in rubber and rubber-like materials. They assumed that Griffith’s approach is valid for the case of large deformation, the irreversible changes in energy due to crack growth take place only in the crack tip vicinity, and the change in energy is independent of the geometry. Therefore, the crack growth is governed by the critical tearing energy criterion that is defined by

1 $$\begin{aligned} T_{\rm {c}} = - \frac{\partial U}{\partial A}\bigg |_{\delta _{\rm {c}}}, \end{aligned}$$

where $T_{\rm {c}}$ is the critical tearing energy, $A$ is the surface area of one face of the crack, $U$ is the potential energy stored in the system and the suffix $\left( \bullet \right) _{\delta _{\rm {c}}}$ denotes that the differentiation is carried out at a constant displacement ${\delta _{\rm {c}}}$, i.e. the external forces do not produce work. The approach was experimentally verified, concluding that the tearing energy vs. rate of tearing relation is a fundamental material property^1,3–5^. Furthermore, the $J$-integral approach^6^ was later extended to rubber and rubber-like materials by Chang^7^ as an alternative approach and the critical $J$-value ($J_{\rm {c}}$) was introduced as equivalence. Moreover, the critical tearing energy has been widely used to study crack initiation and growth in other soft materials such as hydrogels^8^ and fibrous biological tissues^9^.

Several experimental techniques using different specimens were proposed for determining the critical tearing energy. Rivlin and Thomas^1^ introduced the trouser, pure shear, angled and split specimens and since then new specimens have been continuously proposed in the literature, e.g. the single edge notch in tension (SENT)^10^, the double cantilever beam (DCB)^11,12^, tensile strip test^5,13^, the doubly cracked pure shear specimen (DCPS)^14^ and the circumferentially-cracked cylindrical specimen (CCC)^15^. The evaluation of the critical tearing energy is generally accomplished by the determination of the potential decrease due to crack growth, i.e. $\partial U/\partial A$. Analytically, in specimens of simple geometries, the tearing energy is obtained from the energy balance assuming that the decrease in potential energy is due to creation of new crack surfaces^1,11,12,14,15^. Other methods are based on constructing a relation between the total energy stored in the system at the crack initiation and the crack length experimentally using specimens with different initial crack length^10,16–18^. These methods assume a purely elastic material and ignore inelastic deformation effects.

Many experimental investigations reveal that rubber and rubber-like materials experience remarkable microstructural changes during deformation such as the development of cavitation damage, breakage of filler-polymer bonds and crystallisation^19–24^. These processes result in inelastic changes including stress softening (Mullins softening effect^25^), hysteresis, permanent set and induced anisotropy. Thus, a significant amount of the strain energy can be dissipated during deformation which may lead to inaccurate estimation of the critical tearing energy. It is worth mentioning that some soft materials experience similar inelastic changes during deformation such as hydrogels and fibrous biological tissues (e.g. see Long and Hui^8^ and Humphrey^26^). In the context of fracture mechanics, Andrews^27^ showed, in his theoretical study of an infinite inelastic lamina containing a crack, that the inelastic deformation has a significant role in the total energy change due to crack propagation. Early studies attempted to extend the analytical formula of the tearing energy to materials that dissipate energy by assuming that the stored energy density available for crack extension is the recoverable energy density under the unloading curve without exploring the unloading conditions and the self-similarity of the crack tip fields^28–30^. Recently, Qi et al.^31^ proposed theoretical and computational frameworks to study fracture toughness under steady-state crack propagation assuming neo-Hookean solid with rate-independent hysteresis described by the Mullins effect. It is worth mentioning that the determination of intrinsic fracture energy of rubber-like solids—the energy needed to propagate a crack by a unit area without bulk dissipation—is still under investigation^32^. Thus, an accurate estimation of the critical tearing energy might help in the determination of the intrinsic fracture energy.

In the present work, a new method for determining the critical tearing energy in rubber and rubber-like materials is introduced. The critical tearing energy is determined from the actual stored elastic energy such that the effect of dissipated energy due to the inelastic deformation is taken into account. The actual stored elastic energy is estimated experimentally using cyclic pure shear and simple shear dynamic experiments. The unloading rate is experimentally obtained from the time the crack takes to grow an increment. This method can also be used in other soft materials that experience energy dissipation during loading (i.e. manifested in the form of hysteresis) such as hydrogels and fibrous biological tissues.

## Analysis of crack propagation in pure shear tear test

**Figure 1:**
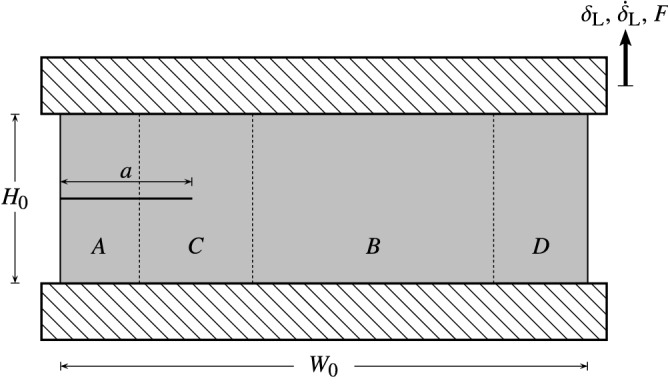
The schematic of the pure shear tear specimen.

Rivlin and Thomas^1^ proposed the pure shear tear test for characterising the tearing energy in rubber-like materials which are assumed to exhibit purely elastic behaviour. In this setup, a pre-cracked pure shear specimen with a low ratio between the height and width is used. A typical specimen is illustrated in Fig. 1 in which the undeformed width, height, and thickness of the specimen are denoted by $W_0$, $H_0$ and $B_0$, respectively, and the crack length by $a$. The loading is defined by the displacement $\delta _{\rm {L}}$ and its rate ${\dot{\delta }}_{\rm {L}}$ on the boundary where the applied force is $F$. Moreover, the critical tearing energy is generally estimated under quasi-static loading conditions (i.e. ${\dot{\delta }}_{\rm {L}} \rightarrow 0$) in which the loading rate effects are minimal.

### The classical method

In order to study the crack propagation, the specimen is divided into four different regions, based on the deformation state: (*i*) region A behind the crack tip in which the material is unloaded, (*ii*) region B is in a state of pure shear deformation, (*iii*) region C, between regions A and B, is in a complicated state of deformation, and (*iv*) region D is between the pure shear region and the traction-free edge. The propagation of the crack is assumed to take place at a fixed separation between the clamps, i.e. $\delta _{\rm {L}} = \mathrm {const.}$, and is seen as a shift of region C in the direction of the propagation. Consequently, region A will increase while region B will decrease by the same amount. Hence, the propagation of the crack by $\mathrm {d}a$ (measured in the undeformed configuration) is a process of unloading a volume of $H_0 \, B_0 \, \mathrm {d}a$ from the pure shear deformation to the undeformed state. The change of the potential energy $\mathrm {d}U$ in the specimen is defined as

2 $$\begin{aligned} \mathrm {d}U = U\left( a+ \mathrm {d}a \right) - U\left( a\right) = - H_0 \, B_0 \, \mathrm {d}a \, \Psi , \end{aligned}$$

where $\Psi$ is the elastically stored energy per unit referential volume of the material in a state of pure shear at the critical displacement $\delta _{\rm {L}} = \delta _{\rm {c}}$ (i.e. at which the crack propagation takes place). It should be noted that the change in the total energy is equal to the change in the elastic stored energy in the case of purely elastic materials. Additionally, the stress-strain state in the vicinity of the crack tip in region C is taken to be self-similar during crack initiation as $\mathrm {d}a \rightarrow 0$. Therefore, using Eq. (1), the critical tearing energy can be determined as

3 $$\begin{aligned} T_{\rm {c}} = \Psi \, H_0. \end{aligned}$$

**Figure 2:**
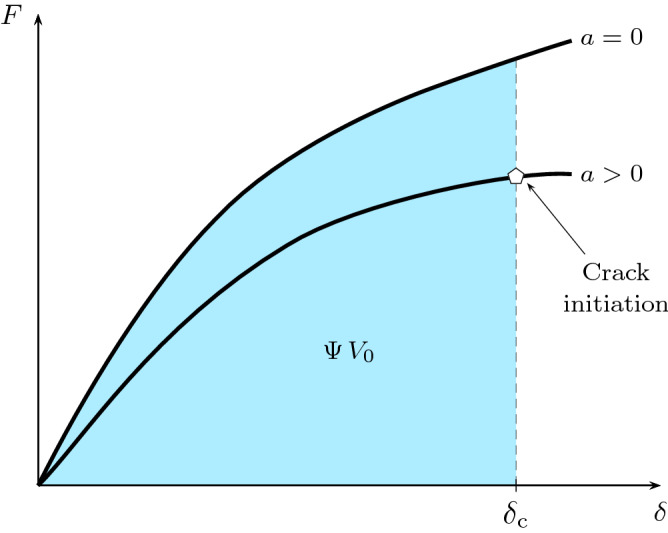
The schematic of the load-displacement curve: the shaded area is the strain energy stored in the uncracked specimen at the critical displacement $\delta _{\rm {c}}$.

The load-displacement relation of a pure shear test under quasi-static loading conditions is assumed to take the general form $F = F\big (\delta _{\rm {L}},a \big )$. Therefore, the stored energy per unit referential volume $\Psi$ is obtained by graphical integration under the load-displacement curve of an uncracked pure shear specimen of the material ($a=0$), see Fig. 2, as

4 $$\begin{aligned} \Psi \big (\delta _{\rm {c}}\big ) = \frac{1}{V_0} \, \int \limits ^{\delta _{\rm {c}}}_{0} F\big (\delta _{\rm {L}},0\big ) \, \mathrm {d}\delta _{\rm {L}}, \end{aligned}$$

where $V_0 = W_0 H_0 B_0$ is the volume of the specimen in the reference configuration. Strictly speaking, this relation is valid under the assumption of an ideal rubber-like solid in which the deformation is assumed to be purely elastic and the load-displacement relation is reversible.

### The modified method

**Figure 3:**
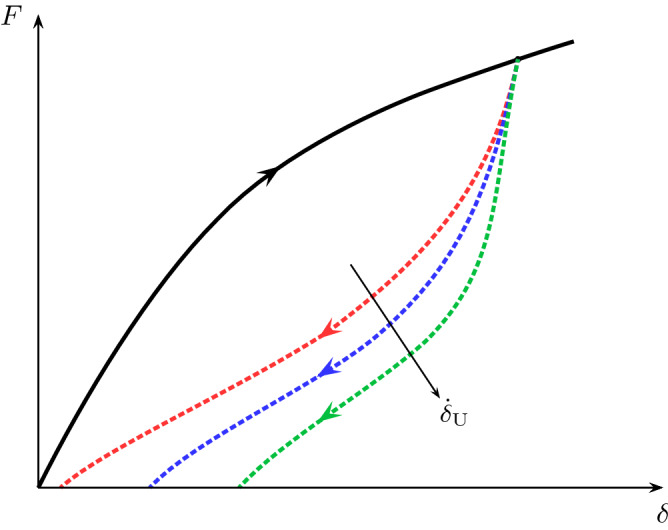
The schematic of the load-displacement curve of a non ideal rubber-like material. The inelastic effects are demonstrated by the irreversibility of the loading-unloading behaviour for different unloading rates ${\dot{\delta }}_{\rm {U}}$.

The load-displacement relation for a non-ideal rubber-like material in the pure shear test is irreversible and may depend on the unloading rate^33^, see Fig. 3. The total change in the total internal energy per unit reference volume of the uncracked pure shear specimen, $\dot{E}$, can be divided into the change in the *elastic* free energy per unit reference volume ${\dot{\Psi }}$, *heat* and *dissipation* energy per unit reference volume $\dot{Q}$, and *free* energy in other forms per unit reference volume $\dot{\Psi '}$ (e.g. free energy stored as surface energy between the amorphous and crystalline phase during the strain-induced crystallisation):

5 $$\begin{aligned} \dot{E} = {\dot{\Psi }} + \dot{Q} + \dot{\Psi '}. \end{aligned}$$

**Figure 4:**
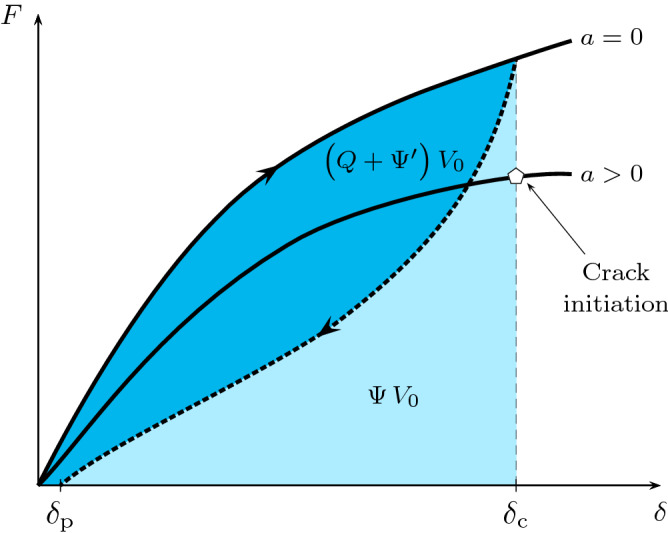
The schematic of the load–displacement curve for a non ideal rubber-like material. The lighter shaded area is the elastic stored energy, $\Psi \, V_{0}$, and the darker shaded area is the summation of the heat and dissipation energy and the free energy in other forms, $\big ({Q} + {\Psi '} \big ) \, V_{0}$, in the uncracked specimen at the critical displacement $\delta _{\rm {c}}$. $\delta _{\rm {p}}$ is the permanent deformation after unloading.

In Fig. 4, the elastically stored energy is the area under the unloading curve and the area between the loading and unloading curves is associated with $Q$ and $\Psi '$. Hence, the actual change in the stored energy due to crack propagation in a pure shear tear specimen is associated with the true elastic energy $\Psi$ rather than the assumed elastic energy.

The irreversible load-displacement relation in a pure shear test can be expressed as $F = F\big (\delta ,{\dot{\delta }},a; {\varvec{\kappa }}\big )$, where $\delta$ and ${\dot{\delta }}$ are the displacement and its rate; and ${\varvec{\kappa }}$ are some internal variables that describe the different inelastic processes and determine the changes in the load-displacement behaviour. The internal variables, ${\varvec{\kappa }}$, are history and rate dependent; and can be determined by a set of evolution laws that may take the general form ${\dot{{\varvec{\kappa }}}} = {\dot{{\varvec{\kappa }}}}(\delta ,{\dot{\delta }},\Theta ,{\varvec{\kappa }})$, where $\Theta$ is the absolute temperature. The actual elastic energy is then obtained by

6 $$\begin{aligned} \Psi \big (\delta _{\rm {c}},{\dot{\delta }}_{\rm {U}} \big ) = \frac{1}{V_0} \, \int \limits ^{\delta _{\rm {p}}}_{\delta _{\rm {c}}} F\big (\delta _{\rm {U}},{\dot{\delta }}_{\rm {U}},0; {\varvec{\kappa }}\big ) \, \mathrm {d}\delta _{\rm {U}}, \end{aligned}$$

and the summation of the heat and dissipation energy becomes

7 $$\begin{aligned} {Q} + {\Psi '} = \frac{1}{V_0} \, \oint \limits ^{\delta _{\rm {p}}}_{0} F\big (\delta ,{\dot{\delta }},0; {\varvec{\kappa }}\big ) \, \mathrm {d}\delta , \end{aligned}$$

where $\delta _{\rm {p}}$ is the permanent deformation after the pure shear specimen is completely unloaded and ${\varvec{\kappa }}$ account for the evolution of inelastic effects during loading and unloading. It should be noted that the forms of the load-displacement and internal variables relations are out of the scope of the current study and we use direct experimental measurements. Further, it is not an easy task to obtain the exact unloading rate of region B during crack growth. Therefore, the unloading rate effect on the recovered elastic energy will be investigated.

The unloading rate can directly be estimated from the experimentally measured crack growth rate. Thus, denoting the time taken by the crack to grow an increment $\Delta a$ by ${t}_{\rm {c}}$, the average unloading rate due crack growth is determined as

8 $$\begin{aligned} {\dot{\lambda }}_{\rm {U}} = \frac{{\lambda }_{\rm {c}}-{\lambda }_{\rm {p}}}{{t}_{\rm {c}}}, \end{aligned}$$

where ${\dot{\lambda }}_{\rm {U}} = {\dot{\delta }}_{\rm {U}}/H_{0}$ is the unloading stretch rate, ${\lambda }_{\rm {c}} = 1+\delta _{\rm {c}}/H_{0}$ is the critical stretch and ${\lambda }_{\rm {p}} = 1+\delta _{\rm {p}}/H_{0}$ is the permanent stretch.

The actual elastic and; heat and dissipation energies, can be estimated using different experiments. One method is to use *cyclic pure-shear experiment*, see Fig. 4. Here, uncracked pure-shear specimens are cyclically loaded from the undeforemed state (i.e. $\delta =0$) to the critical displacement (i.e. $\delta =\delta _{\rm {c}}$) and then are unloaded to zero force (i.e. $F=0$ and $\delta =\delta _{\rm {p}}$). The loading and unloading rates are set to be equal to the rates in the case of cracked pure-shear specimen. It follows that the actual elastic and; heat and dissipation energies are determined graphically from the load-displacement response as in Eqs. (6) and (7), respectively. Another method is to use the *dynamic simple shear experiment* in which the storage and loss shear moduli data, i.e. $G'$ and $G''$, respectively, are determined for a wide range of frequencies. Typically, a harmonic shear strain is applied, i.e. $\gamma \left( t \right) = \gamma _{\rm {a}} \, \cos \left( \omega \, t \right)$, where $\gamma _{\rm {a}}$ is the amplitude and $\omega$ is the frequency in $\mathrm {rad}/\mathrm {s}$, which results in a harmonic shear stress with a phase shift $\tau \left( t \right) = \tau _{\rm {a}} \, \cos \left( \omega \, t + \phi \right)$, where $\tau _{\rm {a}}$ is the shear stress amplitude and $\phi$ is the equivalent loss angle. Hence, the storage and loss moduli are determined from $G' = \tau _{\rm {a}}/\gamma _{\rm {a}}$ and $G'' = G' \, \rm {tan}\left( \phi \right)$, respectively. The dissipated energy during half a cycle (i.e. includes one loading and unloading path) at a given frequency can then be expressed by

9 $$\begin{aligned} {Q} + {\Psi '} = \frac{1}{2} \oint \tau \mathrm {d}\gamma = \frac{1}{2} \pi \, \tau _{\rm {a}} \, \gamma _{\mathrm {a}} \, \mathrm {tan}\left( \phi \right) . \end{aligned}$$

The simple shear is assumed to differ from pure shear only by a rotation^34^, therefore, the test is equivalent to pure shear test taking

10 $$\begin{aligned} \gamma _{\rm {a}} = \lambda - \dfrac{1}{\lambda }, \quad \text {and} \quad \tau _{\rm {a}} = \dfrac{\lambda }{\lambda ^2+1} \, \sigma , \end{aligned}$$

where $\lambda$ and $\sigma$ are the stretch and Cauchy stress in pure shear. Additionally, the relationship between shear strain and stretch rates can directly be obtained by differentiation of Eq. (10) with respect to time. It should be mentioned that the response of simple and pure shear can be equivalent for small range of stretch in the large deformation regime^35^, i.e. $\lambda \le 1.4$. Additionally, the test loading frequency should be similar to the loading and unloading rates of the cracked pure-shear specimen. Thus, the knowledge of the dissipated energy and the total energy from the cracked pure-shear specimen test allows to obtain the actual elastic energy using Eq. (5).

Experimental studies of the deformation field of a stationary crack in rubber-like materials suggest that the deformation field near the crack tip can be characterised by three regions: (*i*) closest to the crack tip where deformation is significantly large and damage takes place; (*ii*) the interim region that contains inhomogeneous moderate deformation; and (*iii*) far from the crack tip which is characterised by homogenous moderate remote deformation^32,36–38^. Therefore, for a very small crack growth increment, the dissipation in the bulk due to unloading is predominantly taking place in the far region. Additionally, self-similarity of the crack tip fields is valid if the increment is very small in comparison with the specimen dimensions, i.e. $\mathrm {d}a/H_{0} \rightarrow 0$.

## Experimental work

**Figure 5:**
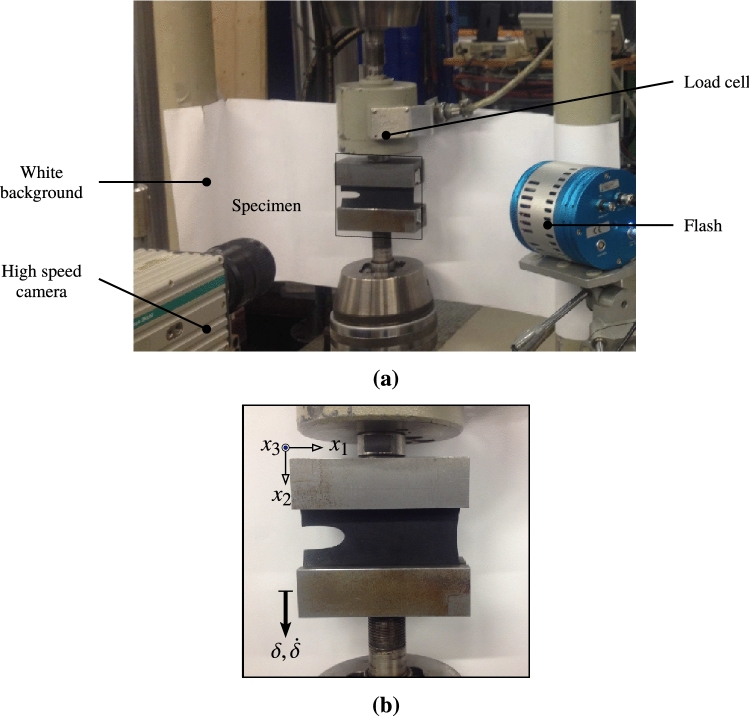
The experimental setup: **(a)** the loading machine, the cracked pure shear specimen and the high speed camera; **(b)** the cracked pure shear specimen, the loading direction and the experiment frame.

A carbon-black-filled natural rubber material is investigated in this experimental study. The material is manufactured by TrelleborgVibracoustic under the designation NR3233 and its chemical properties are listed in Table 1. Two types of specimens have been used in this investigation, i.e. the uncracked and cracked pure-shear specimens. The specimens were of width $W_0=110~\mathrm {mm}$, height $H_0=30~\mathrm {mm}$, and thickness $B_0=2.5~\mathrm {mm}$. In the cracked pure-shear specimens, initial cracks of length $a=30~\mathrm {mm}$ were created using razor blades. A standard servo-hydraulic test machine of load capacity $50~\mathrm {KN}$, was used and the different tests were performed at ambient temperature between $22$-$25^\circ \mathrm {C}$ and relative humidity $60\%$. Additionally, a high speed camera at up to $7000~\mathrm {frames}/\mathrm {s}$ was used to detect the crack propagation onset and to follow the progress of the crack and later a post-processor was used to obtain the crack trajectory and velocity. The experiment setup is shown in Fig. 5.

**Table 1:** The mix formulation in parts per hundred rubber by weight (phr) and Shore A hardness of carbon-black-filled natural rubber (NR3233).

NR	CB	Plasticizer	Additives	Shore A
100	54	13	19	50

Four cracked pure-shear specimens were monotonically loaded at cross head speed of $0.3~\mathrm {mm}/\mathrm {s}$ until complete failure. It is worth noting that, at this loading rate, the material shows very limited rate sensitivity. The load-displacement graphs were recorded and the crack growth points were marked during the test, and the critical displacement was determined. The uncracked pure-shear specimens were subjected to a cycle of loading and unloading. They were monotonically loaded until the critical displacement, i.e. obtained from the cracked pure-shear specimen, was reached and then they were unloaded completely. The loading cross head speed was kept at $0.3~\mathrm {mm}/\mathrm {s}$ ($0.01~\mathrm {s}^{-1}$), as in the case of the cracked specimens. During unloading, the cross head speed was varied to investigate the effect of the unloading rate. Therefore, three unloading cross head speeds were used, i.e. $0.3$, $3.0$ and $30.0~\mathrm {mm}/\mathrm {s}$ ($0.1$, $0.1$, and $1.0~\mathrm {s}^{-1}$). Three specimens per unloading rate were tested such that nine specimens were used in total. In addition to these experiments, dynamic simple shear experimental data are available in Österlöf et al.^39^ for the same material.

## Results and discussion

**Figure 6:**
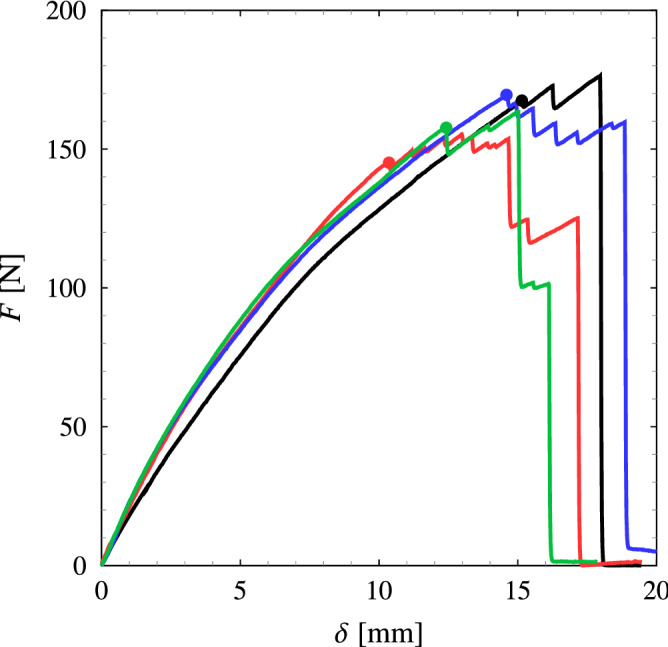
The load-displacement curves of four cracked pure-shear specimens where the solid circles denote the crack initiation points.

Figure 6 shows the load-displacement records of four cracked pure-shear specimens in which the solid circles are the crack initiation points. The average critical displacement is determined to be ${\bar{\delta }}_{\rm {c}} = 13.131~\mathrm {mm}$ with a standard deviation of $1.897~\mathrm {mm}$ which corresponds to the average stretch ${\bar{\lambda }}_{\rm {c}} = 1.44$.

**Figure 7:**
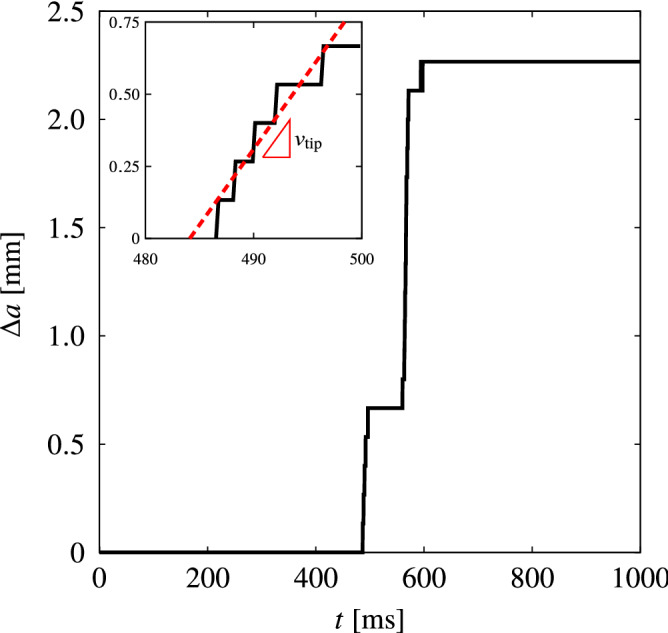
The crack extension vs time.

Figure 7 illustrates the relationship between the crack extension and time at the onset of propagation. The result shows that the crack propagates in a stick-slip fashion with average increment of $\Delta {a} \approx 0.6~\mathrm {mm}$. The average crack velocity is determined to be $\dot{a} = 52~\mathrm {mm}/\mathrm {s}$ with a standard deviation of $2.5~\mathrm {mm}/\mathrm {s}$. Thus, the average crack propagation time over the increment is ${t}_{\rm {c}} \approx \Delta {a}/\dot{{a}} = 11.5~\mathrm {ms}$. The average unloading rate due crack growth is determined as ${\dot{\lambda }}_{\rm {U}} = \left( {\bar{\lambda }}_{\rm {c}}-{\bar{\lambda }}_{\rm {p}}\right) /{t}_{\rm {c}} \approx 125.2~\mathrm {s}^{-1}$ where ${\bar{\lambda }}_{\rm {p}} \approx 1$ is the average permanent stretch that will be discussed in the next paragraph.

**Figure 8:**
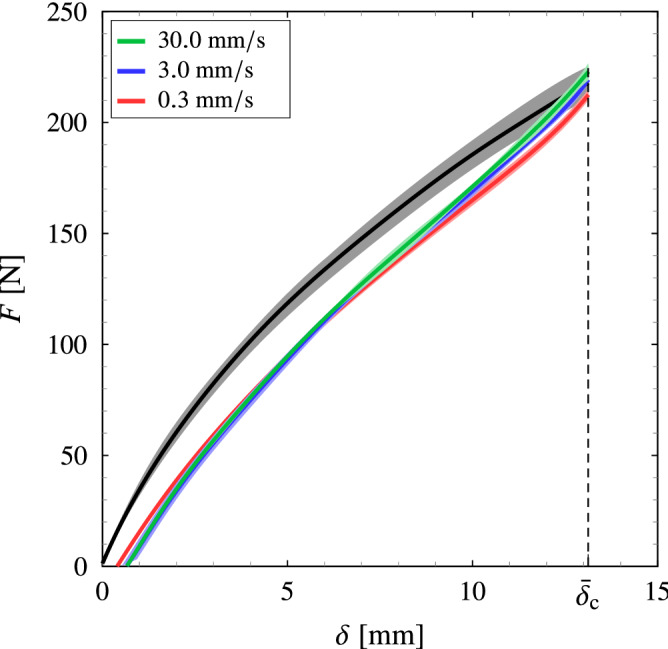
The load–displacement curves of uncracked pure-shear specimens at loading rate of $0.3~\rm {mm}/\mathrm {s}$ ($0.01~\mathrm {s}^{-1}$) and different unloading rates. The red, blue and green lines represent the unloading rates of $0.3$, $3.0$, and $30.0~\mathrm {mm}/\mathrm {s}$ (i.e. $0.01$, $0.1$, and $1.0~\mathrm {s}^{-1}$), respectively, and fill areas represent the experiment scatter (error bars). The unloading point is taken at ${\bar{\delta }}_{\rm {c}} = 13.131~\mathrm {mm}$.

Typical load-displacement records for an uncracked pure-shear specimen are shown in Fig. 8, wherein the specimens are loaded under controlled deformation until the average critical displacement ${\bar{\delta }}_{\rm {c}}$ and then unloaded. The material shows nonlinear large deformation behaviour and significant energy dissipation. The average permanent deformation is determined to be ${\bar{\delta }}_{\rm {p}} = 0.4$, $0.66$ and  $0.66~\mathrm {mm}$ for the unloading rates $0.01$, $0.01$ and $1.0~\mathrm {s}^{-1}$, respectively. Therefore, the result suggests that the permanent stretch is insignificant, i.e. ${\bar{\lambda }}_{\rm {p}} = 1+{\bar{\delta }}_{\rm {p}}/H_{0} \approx 1$.

The total energy $E$ and actual elastic energy $\Psi$ per unit volume were evaluated numerically using the load–displacement records of the uncracked pure-shear specimens at different unloading rates. The critical tearing energy was then determined using the classical and proposed methods using Eqs. (4) and (6), respectively; together with Eq (3). Using the classical method, the critical tearing energy is found to be $\bar{T}_{\rm {c}} = 7.04~\mathrm {kJ}/\mathrm {m}^2$ with a standard deviation of $0.43~\mathrm {kJ}/\mathrm {m}^2$. Table 2 shows the critical tearing energies using the true stored elastic energy at different unloading rates ${\dot{\lambda }}_{\rm {U}}$. The result suggests that the recovered elastic energy weakly depends on the unloading rate for the given experimental range (${\dot{\lambda }}_{\rm {U}} \le 1.0~\mathrm {s}^{-1}$) which indicates that Mullins effect is the dominant contributor. The experimental unloading rate falls outside the experimental unloading rates which requires further investigates.

**Table 2:** The average critical tearing energy $\bar{T}_{\rm {c}}$ and its standard deviation ${\tilde{\sigma }}_{T_{\rm {c}}}$ using the true stored elastic energy for different unloading rates ${\dot{\lambda }}_{\rm {U}}$ from the cyclic pure shear experiment.

${\dot{\lambda }}_{\rm {U}}$ [$\mathrm {s}^{-1}$]	$\bar{T}_{\rm {c}}$ [$\mathrm {kJ}/\mathrm {m}^2$]	${\tilde{\sigma }}_{T_{\rm {c}}}$ [$\mathrm {kJ}/\mathrm {m}^2$]
0.01	6.17	0.49
0.1	6.00	0.54
1.0	5.82	0.48

**Figure 9:**
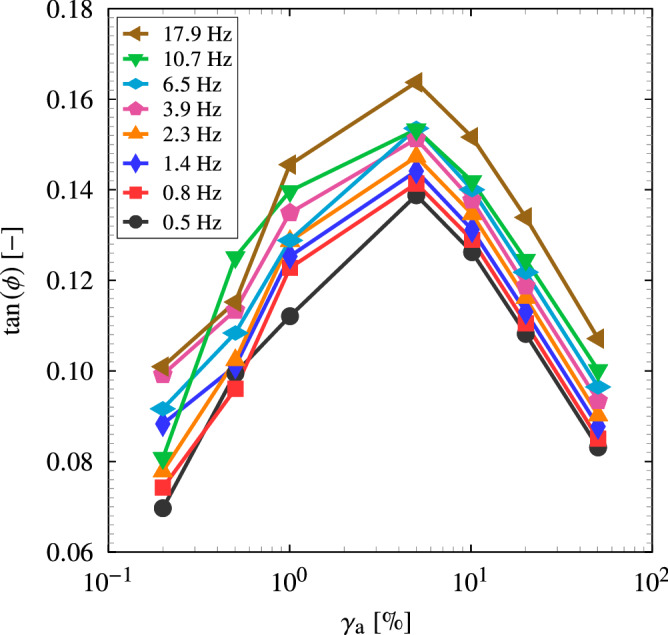
The relationship between the tangent of the loss angle and the shear strain amplitude for different frequencies using simple shear dynamic experiment^39^.

**Figure 10:**
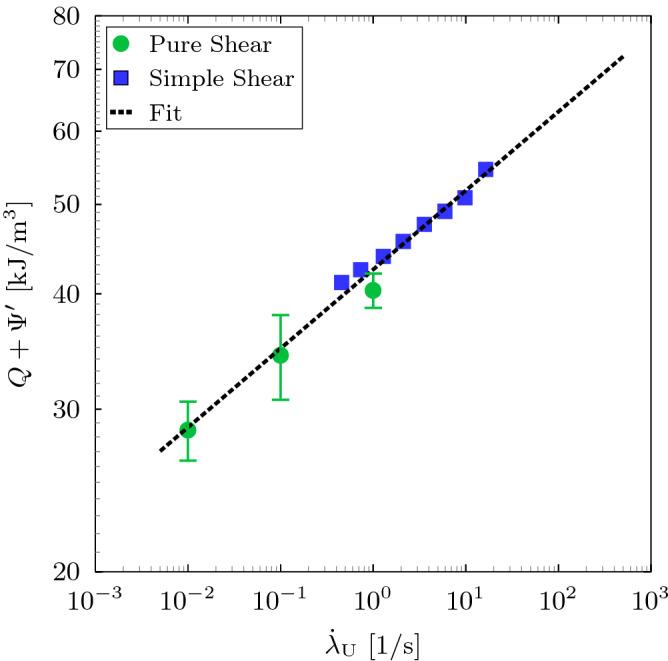
The relationship between the total dissipation and the unloading rate for pure shear and simple shear dynamic experiments.

**Figure 11:**
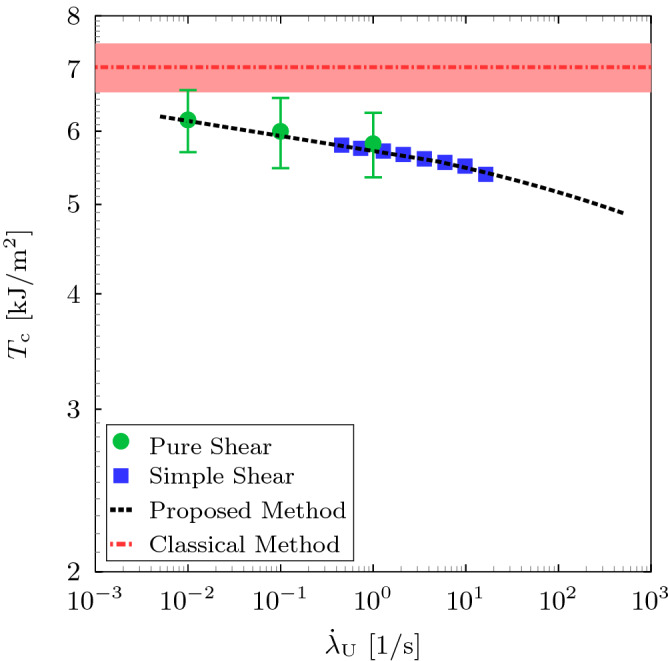
The relationship between the critical tearing energy and the unloading rate for pure shear and simple shear dynamic experiments using the proposed method where the dashed black line is the fitting. The red chain line represents the average tearing energy using the classical method and fill area represents the experiment scatter (error bar).

To determine the dissipation at the experimental unloading rate, we consider the storage and loss shear moduli data available in Österlöf et al.^39^ for the same material and obtained from simple shear experiment with a harmonic excitation. Hence, the loss angle $\phi$ is obtained from the storage and loss shear moduli data^39^, i.e. $\phi = \arctan \left( G''/G'\right)$. Fig. 9 shows the loss angle as a function of the strain amplitude and for different frequencies. The result implies that the dissipation depends on the strain amplitude and frequency. At lower and higher strain amplitudes, the dissipation weakly depends on the frequency which suggests that the dissipation is mainly due to Mullins effect and the viscous dissipation is a minor part. At intermediate strain amplitudes, the viscous dissipation appears to dominate. The equivalent shear strain for the critical stretch $\lambda _{\rm {c}}$ is calculated using Eq. (10) to be $\gamma _{\rm {a}} \approx 0.75$. The shear stress is then calculated using the storage modulus, i.e. $\tau _{\rm {a}} = G' \, \gamma _{\rm {a}}$, and the tangent of the equivalent loss angle is obtained from Fig. 9. Thus, for $\gamma _{\rm {a}} \approx 0.75$, the dissipation can be estimated using Eq. (9) for the range of frequencies. The equivalent average unloading stretch rate can be obtained by ${\dot{\lambda }}_{\rm {U}} = 4 \left( \lambda _{\rm {c}}-\lambda _{\rm {p}}\right) /T$, where $T=2 \, \pi /\omega$ is the periodic time such that the unloading path takes quarter the periodic time. The unloading rates for the frequency range $0.5$-$18~\mathrm {Hz}$ is obtained to be $0.46$-$16.41~\mathrm {s}^{-1}$. It is worth noting that the shear strain rate varies during the simple shear dynamic test and we limit our considerations to the average stretch rate. Additionally, direct differentiation of Eq. (10) with respect to time yields similar stretch rates. Figure 10 illustrates the total dissipated energy as a function of the unloading stretch rate. The result implies that cyclic pure shear and simple shear dynamic tests yield similar outcome where the amount of dissipated energy increases with the unloading rate. The rate sensitivity of the dissipation is due to the increase of viscous contribution. The critical tearing energy as a function of the unloading rate is then calculated using Eq. (3). Figure 11 shows the relationship between critical tearing energy and unloading rate wherein the difference between the classical and proposed methods increases with the increase of unloading rate due to the increase in viscous dissipation. For the crack propagation unloading rate $125.2~\mathrm {s}^{-1}$, using the fitting in Fig. 10, the dissipated energy is $64.23~\mathrm {kJ}/\mathrm {m}^{3}$ which corresponds to $125.4\%$ increase from the lowest unloading rate. Thus, using the fitting in Fig. 11, the average tearing energy becomes $5.12~\mathrm {kJ}/\mathrm {m}^2$ which is $37.5\%$ less than the tearing energy estimated using the classical method.

## Conclusions

In conclusion, a modified method for estimation of the critical tearing energy in rubber-like solids has been presented. The method is a modification of the classical method proposed by Rivlin and Thomas^1^ using the pure shear tear test. In this method, the total energy stored in a rubber-like material is divided into elastic and inelastic contributions taking into account the different inelastic processes. Hence, the energy required for crack propagation is determined by the change of the elastically stored energy only rather that the total energy in the case of the classical method. The true elastic stored energy is determined at unloading rate obtained experimentally from the time the crack takes to grow an increment. A carbon-black-filled natural rubber material is experimentally investigated. The crack growth average increment is significantly smaller than the specimen’s dimensions, i.e. $\Delta {a}/a = \Delta {a}/H_{0} \approx 0.02$, which suggests that the self-similarity of the crack field can be a valid assumption (experimentally observed damage zone in rubber-like materials is about $20\%$ of the crack length^36,37^). The total dissipated energy is evaluated using cyclic pure shear experiment and simple shear dynamic data available in Österlöf et al.^39^ at the critical stretch level. A power-law relationship between dissipated energy and unloading stretch rate is determined using the cyclic pure shear and simple shear dynamic experiments. The result implies that the dissipated energy can be estimated using cyclic pure shear and simple shear dynamic tests. Strictly speaking, to minimise the uncertainty in estimating the total dissipation, it should be obtained using the loading and unloading rates of the cracked pure shear specimen. The analysis of dissipation suggests that, at the critical stretch level, Mullins effect and viscous dissipation contribute to the total dissipation with the viscous contribution increases with the increase of unloading rate. For the crack propagation unloading rate $125.2~\mathrm {s}^{-1}$, the viscous dissipation is significant such that the total dissipation is $\approx 64.23~\mathrm {kJ}/\mathrm {m}^{3}$ (i.e. $125.4\%$ increase in the total dissipation from the lowest unloading rate). The average critical tearing energy becomes $5.12~\mathrm {kJ}/\mathrm {m}^2$ which is $37.5\%$ less than the tearing energy estimated using the classical method. It is worth mentioning that more accurate estimation of the critical tearing energy should be done by considering experiments at the unloading rate $125.2~\mathrm {s}^{-1}$. However, the results presented here give strong indication of the overestimation in the critical tearing energy due to the excellent correlation between the unloading rate and dissipated energy in Fig. 10. Therefore, to accurately measure the critical tearing energy, the recovered elastic energy should be experimentally measured using cyclic pure shear and simple shear dynamic tests which depends on the material, loading rate, crack propagation unloading rate and the critical stretch $\lambda _{\rm {c}}$. The unloading rate should experimentally be determined for the time the crack takes to propagate an increment. Additionally, the simple shear dynamic experiment offers excellent alternative for determination of the dissipation at different frequencies (unloading rates). The proposed method should be used to study other soft materials that experience energy dissipation during loading such as hydrogels and fibrous biological tissues. Additionally, similar approach can be adopted to estimate the actual tearing in specimens of simple geometries such as the trouser and single edge notch in tension (SENT) specimens.
